# Real-time route planning of unmanned aerial vehicles based on improved soft actor-critic algorithm

**DOI:** 10.3389/fnbot.2022.1025817

**Published:** 2022-12-05

**Authors:** Yuxiang Zhou, Jiansheng Shu, Xiaolong Zheng, Hui Hao, Huan Song

**Affiliations:** Xi’an Research Institute of High Technology, Xi’an, China

**Keywords:** deep reinforcement learning, unmanned aerial vehicles (UAV), 2D path planning, local route planning, SAC algorithm

## Abstract

With the application and development of UAV technology and navigation and positioning technology, higher requirements are put forward for UAV maneuvering obstacle avoidance ability and real-time route planning. In this paper, for the problem of real-time UAV route planning in the unknown environment, we combine the ideas of artificial potential field method to modify the state observation and reward function, which solves the problem of sparse rewards of reinforcement learning algorithm, improves the convergence speed of the algorithm, and improves the generalization of the algorithm by step-by-step training based on the ideas of curriculum learning and transfer learning according to the difficulty of the task. The simulation results show that the improved SAC algorithm has fast convergence speed, good timeliness and strong generalization, and can better complete the UAV route planning task.

## Introduction

Route planning is a special column of path planning, which aims to find the path from the current position to the target position. The path should be as short as possible, the smoothness should fit the flight conditions of the aircraft, and it must be safe and collision-free (Han and Seo, 2017). Due to better mobility and flexibility, unmanned aerial vehicles (UAV) are increasingly used to perform complex and changeable tasks in the flight environment, such as battlefield attack tasks, and post-disaster search and rescue tasks. The research at home and abroad mainly focuses on the route planning of the UAV in a fixed static environment. Therefore, the ability of the UAV to conduct real-time maneuvering obstacle avoidance after acquiring dynamic environmental information becomes particularly important (Park and Baek, 2020).

According to the understanding of the environment in the planning process, path planning can be divided into global path planning and local path planning (Li and Chou, 2018). Traditional optimization algorithms are mostly global path planning algorithms, such as Dijkstra (1959), A * algorithm (Hart et al., 1968), RRT * algorithm (Karaman and Frazzoli, 2011), particle swarm algorithm (Shao et al., 2020) and ant colony algorithm (Liu et al., 2017). Most of the global path planning algorithms apply to route optimization in static and fixed scenes, and a large amount of environmental map information needs to be stored in advance. With the increase of complexity and uncertainty of the environment and the influence of noise, its reliability and applicability will be greatly reduced (He et al., 2021).

Local path planning is to detect the moving environment through sensors to obtain the location and geometric properties of obstacles. This kind of planning requires the real-time collection of environmental data, and the dynamic update of the environmental model can be corrected at any time, which has high robustness to environmental errors and noise, and the planning speed is fast, so it is usually used for real-time route planning (Gao et al., 2020). This method combines the modeling and searching of the environment and requires the robot system to have high-speed information processing and computing capabilities. The artificial potential field algorithm (Khatib, 1985) is a commonly used method in the local path planning algorithm, and its convergence rate is fast. However, the action of the algorithm is discrete, and the planned path smoothness is poor. Path smoothing is usually required before it can be used in actual flight. However, local path planning is only for the actions performed in the current state and does not consider the impact of the actions on the long-term returns of the path. Therefore, although such algorithms have better real-time performance, they often fail to achieve the global optimum of the path.

The deep reinforcement learning algorithm is based on the current state of the environment and takes into account the maximum long-term payoff, so it has good real-time performance and can achieve the overall optimal trajectory, which is superior in solving the online trajectory planning problem. In 2013, the DeepMind team (Mnih et al., 2013) used the fitting function of the neural network to fit the observed high-dimensional environmental data into the Q function, and innovatively proposed the Deep Q-Network (DQN) model, which solved the problem of high-dimensional continuous state space representation, and made deep reinforcement learning become a research hotspot in the field of artificial intelligence. Compared with the traditional algorithm, the reinforcement learning algorithm has better generalization, stronger adaptability to the dynamic changing environment, and better meets the real-time requirements of the online route planning problem. Unlike supervised learning, reinforcement learning automatically acquires sample data during training. In recent years, scholars have combined reinforcement learning with other algorithms to improve the performance of the algorithm, and have achieved good results in offline path planning, online path planning and multi-agent navigation. Lei et al. (2018) used the Double DQN for dynamic path planning with local targets and laser radar detection signals as input. The experimental results show that the algorithm has good generalization and can solve the problem of dimension disaster, but because the algorithm is discrete action space, the smoothness of the planned path is poor. Yu et al. (2020) used a neural network to perceive the environment and extract feature information, and combined DDPG with hierarchical reinforcement learning for path planning. The convergence time was significantly shortened, and the path smoothness was significantly improved. De Jesus et al. (2021) also used a laser radar detection signal as input, compared SAC with DDPG, and concluded that Soft Actor-Critic (SAC) algorithm is more efficient than Deep Deterministic Policy Gradient (DDPG) algorithm. However, the authors did not compare the real-time performance of the two, and the detection range of lidar must be large enough, otherwise, the algorithm will be difficult to converge. Grando et al. (2022) added Recurrent Neural Network (RNN) to Twin Delayed Deep Deterministic policy gradient (TD3) algorithm and SAC algorithm, respectively, so that the model has a certain memory and reasoning ability, and can better avoid obstacles by referring to the preorder information. The experimental results prove the effectiveness of the improved algorithm, and the improved SAC algorithm has faster convergence speed and better effect.

Deep reinforcement learning algorithms are more widely used than traditional reinforcement learning algorithms, and the commonly used algorithms with better convergence effects are based on the deformation of the Actor-Critic structure, such as PPO (Schulman et al., 2017), TD3 (Fujimoto et al., 2018) and SAC (Haarnoja et al., 2018) algorithm. The PPO algorithm is an improved algorithm of the policy gradient algorithm, which was chosen as the default reinforcement learning algorithm by the OpenAI team due to its high adaptability, stable convergence effect and low influence by hyperparameters. The TD3 algorithm is based on the DDPG algorithm and uses a double Q network for estimation, which solves the problem of Q network overestimation and has a more stable convergence effect. In addition, the SAC algorithm is a maximum entropy reinforcement learning algorithm, which enhances the exploration ability of the algorithm and makes it easier for the algorithm to find a better action and prevent the algorithm from falling into a local optimal solution. Therefore, three algorithms are trained and compared in this paper.

The main work of this paper is as follows:

• Based on the UAV real-time route planning problem and the characteristics of deep reinforcement learning algorithm, the formulae for calculating the attractive potential field and repulsive potential field of the artificial potential field algorithm are improved, the potential energy information of the artificial potential field is input into the neural network as part of the state information of the agent for training, and the reward term for the amount of potential energy change is set in the reward function accordingly, which successfully solves the reward sparsity problem arising in the application of reinforcement learning algorithm, and at the same time speeds up the convergence speed and stability of the algorithm and improves the optimization effect.
• In order to prevent the algorithm from generating the phenomenon that it is difficult to train because of the difficulty of the task, this paper combines the methods of curriculum learning and transfer learning to set up a multi-stage training environment by difficulty coefficient for step-by-step training, which improves the training effect of the algorithm, and designs two testing environments to verify the generalization and reliability of the algorithm.

## Deep reinforcement learning algorithm design

### Soft actor-critic algorithm

Soft actor-critic (SAC) algorithm is a model-free random strategy deep reinforcement learning algorithm proposed by Haarnoja in 2018. The rest of this section provides a summary of their work. Its structure includes 1 Actor-network and 4 Critic-networks. The traditional reinforcement learning algorithm only considers maximizing the cumulative reward term, while SAC algorithm maximizes the entropy term of the cumulative reward term and the strategy distribution at the same time. The greater the entropy value is, the greater the randomness of the action is. It reduces the sampling complexity and improves the exploration ability and robustness of the algorithm, preventing premature convergence of the algorithm and generating local optimal solutions. The equation can be written as


$$\pi^{*}=\arg\max_{\pi }{𝔼}_{\left(s_{t},a_{t}\right)∼\rho_{\pi }}[\sum_{t}\underset{\text{reward}}{\underbrace{R\,\left(s_{t},a_{t}\right)}}$$

(1) $$+\alpha_{H}\underset{\text{entropy}}{\underbrace{H\left(\pi \left(\cdot |s_{t}\right)\right)}}].$$

where *r*(⋅) is the Reward value items under the current state and action, *ℋ*(⋅) is the entropy term of strategy π, α is the temperature coefficient. The relative importance of entropy terms of policy distribution is determined by controlling the size α_*H*_.

**FIGURE 1:**
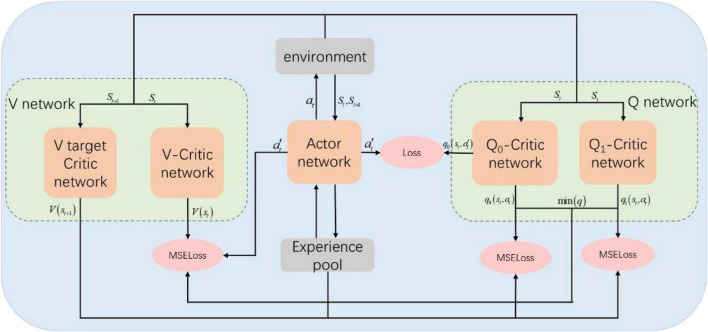
SAC algorithm network architecture.

The SAC algorithm network structure is shown in Figure 1. The parameter of Actor-network is ϕ. The probability distribution π_ϕ_(⋅ | *s*_*t*_) of the strategy is obtained according to the input state *s*_*t*_, and then the action *a*_*t*_ is output according to the probability. The action *a*_*t*_ will be acted on the environment to obtain *s*_*t* + 1_ and *r*_*t* + 1_. The experience data (*s*_*t*_,*a*_*t*_,*r*_*t* + 1_,*s*_*t* + 1_) is stored in the experience pool. The input of the Critic-network is state *s*_*t*_, where the parameter of V network is ψ, and the estimated value of output state *V*_ψ_(*s*); The parameter of Q-network is θ, and the output is state-action value estimation *Q*_θ_(*s*_*t*_,*a*_*t*_).

The mean square error (MSE) loss function of the V-Critic network update can be written as


$$J_{V}\,\left(\psi \right)={𝔼}_{s_{t}∼𝒟}$$

(2) $$\left[\frac{1}{2}\,{\left(V_{\psi }\,\left(s_{t}\right)-{𝔼}_{a_{t}∼\pi_{\phi }}\,\left[Q_{\theta }\,\left(s_{t},a_{t}^{\prime }\right)-\log\pi_{\phi }\,\left(a_{t}^{\prime }|s_{t}\right)\right]\right)}^{2}\right].$$

where *D* is the experience pool.

The gradient can be written as:


$${\hat{\nabla }}_{\psi }\,J_{V}\,\left(\psi \right)=$$

(3) $$\nabla_{\psi }V_{\psi }\,\left(s_{t}\right)\,\left(V_{\psi }\,\left(s_{t}\right)-Q_{\theta }\,\left(s_{t},a_{t}^{\prime }\right)+\log\pi_{\phi }\,\left(a_{t}^{\prime }|s_{t}\right)\right).$$

The gradient here is unbiased estimation, D is the empirical data sample in the empirical pool, and $a_{t}^{\prime }$ is the actor-network generated according to the current state *s*_*t*_. The parameters of the two Q-Critic networks updated by the random gradient descent method are different, and the minimum values of the two *Q*_θ_ are calculated here to show the indigenous accelerated training.

The update of the Q-Critic network is also to minimize the MSE loss function. The equation can be written as


(4) $$J_{Q}\,\left(\theta \right)={𝔼}_{\left(s_{t},a_{t}\right)∼𝒟}\,\left[\frac{1}{2}\,{\left(Q_{\theta }\,\left(s_{t},a_{t}\right)-\hat{Q}\,\left(s_{t},a_{t}\right)\right)}^{2}\right],$$

(5) $$\hat{Q}\,\left(s_{t},a_{t}\right)=r\,\left(s_{t},a_{t}\right)+\gamma \,{𝔼}_{s_{t+1}∼p}\,\left[V_{\bar{\psi }}\,\left(s_{t+1}\right)\right].$$

The gradient can be calculated as


$${\hat{\nabla }}_{\theta }\,J_{Q}\,\left(\theta \right)=$$

(6) $$\nabla_{\theta }Q_{\theta }\,\left(a_{t},s_{t}\right)\,\left(Q_{\theta }\,\left(s_{t},a_{t}\right)-r\,\left(s_{t},a_{t}\right)-\gamma \,V_{\bar{\psi }}\,\left(s_{t+1}\right)\right).$$

The Actor-network is updated by minimizing KL divergence, and the equation can be written as


$$J_{\pi }\,\left(\phi \right)=$$

(7) $${𝔼}_{s_{t}∼𝒟}\left[{\text{D}}_{\text{KL}}\left(.\pi_{\phi }\left(\cdot |s_{t}\right)∥\frac{\exp\left(Q_{\theta }\,\left(s_{t},\cdot \right)\right)}{Z_{\theta }\,\left(s_{t}\right)}\right)\right].$$

where *Z*(⋅) function is used to normalize the distribution.

The strategy is represented as a neural network with noise by the re-parametric technique. It can be written as


(8) $$a_{t}=f_{\phi }\,\left(\epsilon_{t};s_{t}\right).$$

In the formula, _ϵ *t*_ is the noise vector, so the original objective function can be rewritten as


$$J_{\pi }\,\left(\phi \right)=$$

(9) 𝔼st∼𝒟,∼t𝒩[logπϕ(fϕ(;tst)|st)-Qθ(st,fϕ(;tst))].

The gradient is calculated as


$${\hat{\nabla }}_{\phi }\,J_{\pi }\,\left(\phi \right)=\nabla_{\phi }\log\pi_{\phi }\,\left(a_{t}|s_{t}\right)$$

(10) $$+\left(\nabla_{a_{t}}\log\pi_{\phi }\,\left(a_{t}|s_{t}\right)-\nabla_{a_{t}}Q\,\left(s_{t},a_{t}\right)\right)\,\nabla_{\phi }f_{\phi }\,\left(\epsilon_{t};\,s_{t}\right).$$

### Network structure design

The input of reinforcement learning state information requires concise and efficient, and too much useless information in the state information may cause the reinforcement learning algorithm to learn spurious decision correlations and make the deep neural network overfitting, resulting in a significant loss of performance of the algorithm. Therefore, to make the training more efficient, the environmental information collected by the detection device needs to be filtered and processed to better model the correlation between state information and cumulative returns and further make better decisions. In general, the use of relative state information leads to a stronger generalization of the trained algorithm, which enables the direct migration of strategies in similar tasks.

**FIGURE 2:**
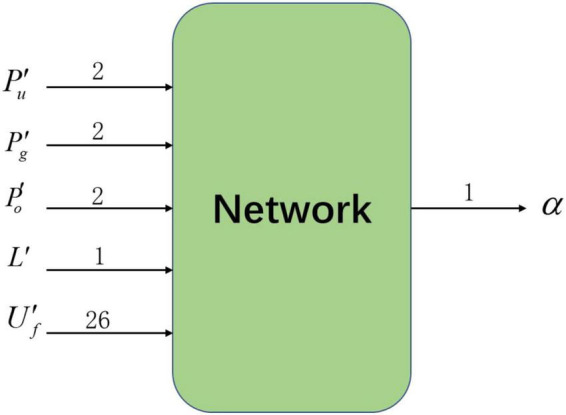
Input and output of the neural network.

As shown in Figure 2, the deep reinforcement learning algorithm used in this paper includes 33 inputs of state information and 1 action output. Among them, the state information input to the neural network is obtained from the data collected by the detection device after filtering and abstraction, including four sets of relative state information: target relative position $P_{g}^{\prime }$, nearest obstacle relative position $P_{o}^{\prime }$, agent relative position $P_{a}^{\prime }$ and range ratio *L*′, and one set of local potential field information $U_{f}^{\prime }$. The output of the neural network is the action chosen by the agent in the current environmental state: turn angle α.

**FIGURE 3:**
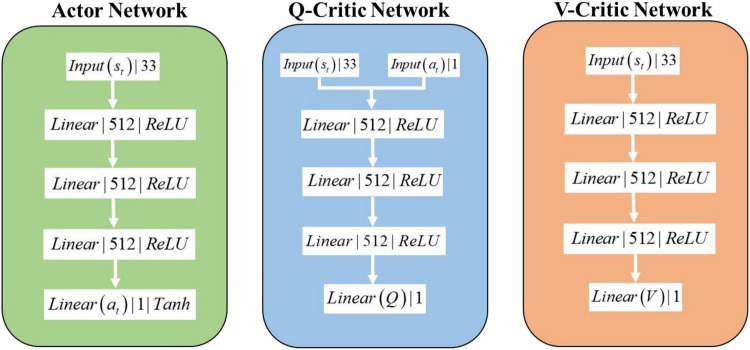
SAC algorithm network structure.

The network structure of the SAC algorithm consists of 1 Actor network, 2 Q-Critic networks with the same structure, and 2 V-Critic networks with the same structure (target network, estimation network), and the inputs and outputs of the network are shown in Figure 3. The hidden layer structure of the Actor network, Q-Critic network and V-Critic network are the same, all containing 3 hidden layers, each with 512 nodes. The input of the Actor network is *s*_*t*_ the current state of the environment where the agent is located, and the output is the turn angle α. The input of the Q-Critic network is *s*_*t*_ and action *a*_*t*_, and the output is the Q value of the current state-action pair. The input of the V-Critic network is *s*_*t*_, and the output is the current state value *V*(*s*_*t*_), which is the value prediction of the current state *s*_*t*_.

## Real-time route planning for unmanned aerial vehicles based on improved soft actor-critic algorithm

### Artificial potential field method

The artificial potential field method is a virtual force method proposed by Khatib (1985), which is widely used to solve the path planning problem of the unmanned system. Its principle is to transform the motion of the unmanned system in the environment into an abstract artificial potential field. Among them, the attractive potential field is generated by the target, which has an attractive effect on the unmanned system and guides the unmanned system to reach the target position quickly. The attractive potential field near the target point is small, and the attractive potential field far from the target point is large. The unmanned system moves in the direction of the negative gradient of the potential field. In the traditional artificial potential field method, the size of the attractive potential field is proportional to the square of the target distance, so that the gravity increases linearly with the target distance. The formula is


(11) $$U_{\text{att}}^{\prime }\,\left(q\right)=\frac{1}{2}\,k_{\text{att}}\,\rho \,{\left(q,q_{\text{goal}}\right)}^{2}.$$

However, the traditional attractiveness formula is insufficient. When the environmental space is large, the gravity of the point far from the target will be much larger than that near the target, resulting in the phenomenon that the unmanned system is prone to hovering near the target point. Therefore, this paper improves the original gravity formula to make the size of the attractive potential field proportional to the target distance. The attractive potential is calculated as


(12) $$U_{\text{att}}\,\left(q\right)=\frac{1}{2}\,k_{\text{att}}\,\rho \,\left(q,q_{\text{goal}}\right).$$

where *k*_att_ is the gain coefficient of attractive potential field, ρ(*q*,*q*_goal_) is the Euclidean distance between the point and the target point.

The repulsion potential field is generated by obstacles, which has a repulsion effect on the unmanned system. The magnitude of repulsion is the negative gradient of the repulsion potential field. The closer the position point is to the obstacle, the larger the repulsion potential field is. In the design of the traditional repulsive potential field formula, within the influence range of obstacles, the size of the repulsive potential field increases sharply with the reduction of the obstacle distance, that is, the size of the repulsive force increases sharply to make the unmanned system away from the obstacles and is not easy to adjust. The repulsion formula is


(13) $${U^{\prime }}_{\text{rep }}(q)=\{\begin{array}{ll} \frac{1}{2}k_{rep}{\left(\frac{1}{\rho \left(q,q_{\text{obs}}\right)}-\frac{1}{\rho_{0}}\right)}^{2}, & if\rho \left(q,q_{\text{obs}}\right)\leq \rho_{0}\\ 0 & if\rho \left(q,q_{\text{obs}}\right)>\rho_{0} \end{array}$$

where *k*_*rep*_ is the gain coefficient of the repulsion potential field, ρ(*q*,*q*_obs_) is the distance between the location point and the obstacle boundary, ρ_0_ is the maximum impact range of obstacles. The repulsive potential field only affects the position points within the maximum influence range.

In this paper, combined with the characteristics of the reinforcement learning algorithm, the exponential function is used to improve the formula of the repulsion potential field, so that the initial value and the change rate of the repulsion potential field can be controlled. The equation can be written as


(14) $$U_{rep}(q)=\{\begin{array}{ll} \alpha_{rep}\cdot e^{-\beta_{rep}\left(\rho \left(q,q_{\text{obs}}\right)-\rho_{0}\right)}, & if\rho \left(q,q_{\text{obs}}\right)\leq \rho_{0}\\ 0 & if\rho \left(q,q_{\text{obs}}\right)>\rho_{0} \end{array}$$

where α_*rep*_ is the initial amplitude adjustment factor of the repulsion potential field, β_*rep*_ is the adjustment factor of the change rate of repulsion potential field size.

The total potential energy of a point is the sum of the attractive potential energy of the target point at that position and the repulsion potential energy of each obstacle at that point, and it can be calculated as


(15) $$U_{a\,l\,l}\,\left(q\right)=U_{a\,t\,t}\,\left(q\right)+U_{r\,e\,p}\,\left(q\right).$$

### State space setting based on potential energy observation

The state space is obtained by the agent’s observation of the environment, which is the basis for the agent’s action selection and includes four parts. In this paper, we introduce the potential energy information of the flight environment and use the potential energy information as a guide to motivate the agent to reach the goal as soon as possible.

#### Relative position of the agent in the environment $P_{u}^{\prime }$

The dimension of the relative position of the agent in the environment is the same as that in the environment, and the value range of each dimension is [0,1], which represents the relative relationship between the agent and the known boundary. Since the environment space is limited, the observed value can prevent the agent movement from exceeding the boundary. In this paper, the rectangular environment, the state of the calculation formula is


(16) $$P_{u}^{\prime }=\frac{P_{u}}{M_{x,y}}.$$

where *P*_*u*_ is the position coordinate of the agent in the original coordinate system, $P_{u}^{\prime }$ is the position coordinate of the agent in the aircraft coordinate system, *M*_*x*_ and *M*_*y*_ represent the length and width of the environment, respectively.

#### Relative position relationship of the target point $P_{g}^{\prime }$

**FIGURE 4:**
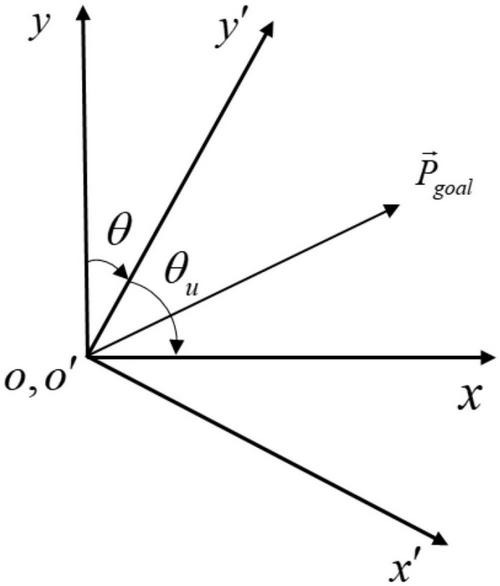
Coordinate transformation diagram.

Taking the target position information in the aircraft coordinate system *x*′*o*′*y*′ as input will be more conducive to the relative relationship between algorithm learning and target points. After the translation and rotation changes of the coordinate system, the original position coordinates of the target point are transformed into the coordinate system with the UAV as the origin, the flight direction of the UAV is the _*y’*_ axis, and the horizontal and vertical direction of the *y*′ axis is the *x*′ axis, as shown in Figure 4. Finally, the numerical normalization is carried out so that the value range of each dimension of $P_{g}^{\prime }$ is [-1,1].

The solving steps are as follows:


(17) $$P_{t\,r\,a\,n}=P_{g\,o\,a\,l}-P_{u}.$$

Firstly, the origin of the original coordinate system is moved to the center of gravity of the UAV through the coordinate system translation transformation, and it can be calculated as

Secondly, the angle of the UAV heading is calculated as θ_*u*_, and the angle to be rotated in the environment coordinate system (clockwise is positive, counterclockwise is negative) is calculated, and the position coordinates of the target point are transformed to the vehicle coordinate system *x*′*o*′*y*′ by the rotation matrix. It can be calculated as


(18) $$\{\begin{array}{c} \theta =\frac{\pi }{2}-\theta_{a}\\ A=\left[\begin{array}{cc} \cos\theta & -\sin\theta \\ \sin\theta & \cos\theta \end{array}\right]\\ P_{r\,o\,t}^{T}=A^{*}\,P_{t\,r\,a\,n}^{T} \end{array}.$$

where θ_*u*_ is the heading angle of the aircraft and matrix _*A*_ is the rotation matrix.

Finally, the coordinate data is calculated as


(19) $$P_{g}^{\prime }=\frac{P_{r\,o\,t}}{\sqrt{M_{x}^{2}+M_{y}^{2}}}$$

#### Relative position relationship of recent obstacles $P_{g}^{\prime }$

Excessive environmental information is not conducive to the learning of the algorithm, and there are many obstacles to the environment. In this paper, only the coordinate position information of the obstacle closest to the agent in the aircraft coordinate system is input. The calculation method is the same as $P_{g}^{\prime }$, and the value range of each dimension is still [-1,1].

#### Relative range information *L*′

The flight range of the UAV is constrained by the maximum flight range *L*_*max*_. Taking the ratio of the flight range information to the maximum flight range as the input can prevent the aircraft from failure due to excessive flight range or long flight time and approach the target area more quickly. It can be written as


(20) $$L^{\prime }=\frac{\sum l_{i}}{L_{\max}}.$$

where *l*_*i*_ is the minimum step size of the UAV flight.

#### Potential field information $U_{f}^{\prime }$

In reinforcement learning algorithms, the dimension of input observation cannot be too large, otherwise it will lead to neural network learning difficult, and it is difficult to extract useful information from input. However, it is difficult to obtain the optimal decision results with less input information, and the algorithm will be difficult to converge. Therefore, it is necessary to take a suitable number of probe points [*p*_1_,*p*_2_,⋯,*p*_*n*−1_,*p*_*n*_] near the location point of the agent. The improved potential energy calculation method is used to calculate the potential energy value *U*_*f*_ of each detection point, and then the obtained potential field information data are normalized by Formula 21 to obtain the potential energy observation data $U_{f}^{\prime }$ with the value range of [0, 1], which is used as part of the input algorithm of the observation data for training. It can be calculated as


(21) $$U_{f}^{\prime }=\frac{U_{i}-U_{\min}}{U_{\max}-U_{\min}}$$

**FIGURE 5:**
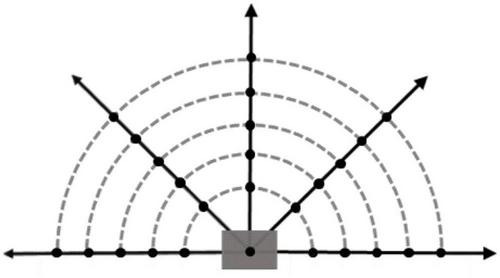
Potential field detection model.

The Fixed-wing UAV can only move forward, so based on the flight direction of the UAV, the environmental potential field information in the range is more useful for the UAV. Taking into account the calculation speed and training effect of the algorithm, such as Figure 5, in this range, five detection directions are taken at equal angles, five detection points are taken at equal intervals in each detection direction, and a position point is added to a total of 26 potential field detection points. In this paper, the interval distance of potential detection points is set equal to the step size of the UAV.

### Action space

**FIGURE 6:**
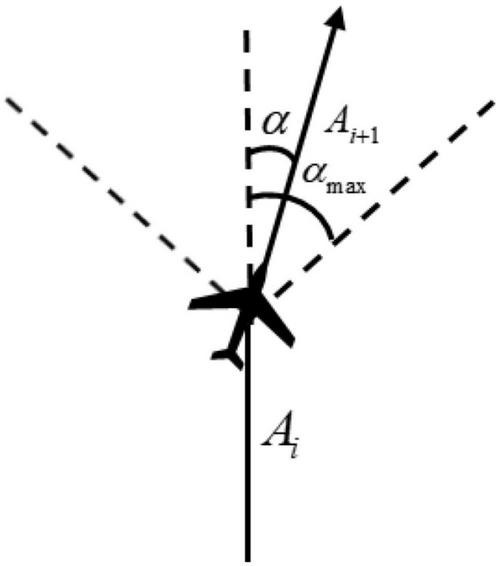
Diagram of turning angle.

The research object of this paper is the fixed-wing UAV, so there is no backward motion in the flight process. In order to make the experiment consistent with the actual situation as much as possible, the action space is designed as continuous action, and the control quantity is the angle of the UAV at the track point. Affected by its own aerodynamic characteristics, the horizontal turning angle of the UAV at each track point cannot exceed the limit of the maximum turning angle. Otherwise, it will lead to instability of the aircraft serious consequences. As shown in Figure 6, the actual turning angle of the aircraft is limited by the maximum turning angle α_*max*_.

Assuming that α_*max*_ is known, *A*_*i*_ is the direction vector of track segment i on the x and y axes, and its expression is *A*_*i*_ = (*x*_*i*_, *y*_*i*_),(*i* = 1, 2,…, *N*), then the relationship between the actual turning angle α and α_*max*_ of UAV is written as


(22) $$\left\{ \begin{array}{c} \alpha_{i}=\frac{A_{i}^{T}\cdot A_{i+1}}{||A_{i}||\cdot ||A_{i+1}||}.\\ -\alpha_{\max}\leq \alpha_{i}\leq \alpha_{\max} \end{array}\right.$$

Within the maximum turning angle limit, the smaller the turning angle of the UAV is, the better the smoothness of the flight trajectory is, but the maneuverability will deteriorate accordingly. Therefore, considering all kinds of factors, the limit of turning angle is [−^15°^,^15°^].

### Reward function setting based on the potential difference

The reward function setting of reinforcement learning mainly needs to solve the problem of sparse reward, which widely exists in practical applications. Sparse reward refers to the agent is difficult to obtain a positive reward in the process of exploration, resulting in low efficiency of algorithm learning and difficult to explore the predetermined state. The reward function is divided into three parts, and the continuous reward is set to guide the UAV to quickly reach the target point with the change of potential energy to solve the problem of sparse reward.

#### Potential energy difference reward *R*_*pf*_

The UAV in this paper moves to the target point under the combined action of target point gravity and obstacle repulsion. Considering the safety factors, the fastest flight direction for the UAV to reach the target is the fastest decline direction of the total force field. Therefore, the potential energy difference reward of the two positions before and after the agent is used as a part of its reward function.

Firstly, according to the improved artificial potential field formula, the position potential energy *U*_*t*_ before action *a*_*t*_ execution and the position potential energy *U*_*t* + 1_ after action *a*_*t*_ execution are obtained, respectively. Then, according to the following formula, the potential energy difference reward *R*_*pf*_ is obtained. It can be calculated as


(23) $$R_{p\,f}=\Delta \,U_{t}=U_{t+1}-U_{t}$$

#### Arrival reward *R*_ar_

For the convenience of calculation, the target is set as a circular target in this experiment, and the target radius is *r*_*g*_. When the distance *d*_*g*_ between the agent and the target center is less than *r*_*g*_, i.e.,*d*_*g*_(*t*) < *r*_*g*_, give a positive reward *R*_ar_.

#### Death penalty *R*_de_

Unmanned aerial vehicles (UAV) threat sources include static fixed obstacles, early warning detection radar and air defense weapons. Similar to target processing, obstacles are treated as two-dimensional circular obstacles. The radius of the threat circle *r*_*o*_ is the maximum radius of the obstacle, the maximum warning detection range of the early warning detection radar and the maximum attack radius of the air defense weapon. When the distance between the agent and the center of the threat source is less than the radius of the threat source, when the agent movement exceeds the maximum range or when the agent movement touches the environment boundary, a negative reward is given.

#### Total reward value *R*_all_

The total reward for the UAV flight is the sum of the potential difference reward, arrival reward and death penalty minus baseline reward *R*_0_ (baseline), as shown in formula 24. By adding a baseline to make each step reward positive or negative, improve the sampling probability of excellent actions. It can be calculated as


(24) $$R_{a\,l\,l}=R_{p\,f}+R_{a\,r}+R_{d\,e}-R_{0}.$$

## Training design based on course learning

### Curriculum learning

Curriculum learning (Bengio et al., 2009) is a training strategy proposed by Bengio. It imitates the learning process of human beings, accelerates learning by setting courses with different degrees of difficulty, and migrates strategies from simple problems to complex problems. This method is widely used in computer vision and natural language processing to improve the generalization ability and training efficiency of various models (Wang et al., 2021).

In the design of the reinforcement learning algorithm, parameter migration is used to migrate the model parameters trained in the previous training environment to the current training environment, and multi-scenario learning is carried out according to the degree of task difficulty. The improved algorithm is verified and compared. In this paper, the training environment of UAV path planning is divided into several different environments corresponding to different training tasks. The first training environment is an open free-motion space, which aims to make the UAV find the nearest path to the target. The second training environment is the space containing obstacles. In this environment, UAV gradually learns to avoid obstacles and find the nearest path to the target.

### Training parameters design

#### Flight parameters

In the reinforcement learning algorithm, each time interval Δ*t*of the agent corresponds to an action, which indicates the completion of a timestep. In this paper, we set the flight speed *V* of the UAV and the time interval Δ*t*of track search to be constant. The UAV does a constant step of track search, which is set to 1.

#### Reinforcement learning related parameters

The deep reinforcement learning algorithm built on PyTorch was used for training optimization and testing, and the parameters of the algorithm were set as shown in Table 1.

**TABLE 1:** Parameter table of deep reinforcement learning algorithm.

Parameter	PPO	SAC	TD3
Learning_rate	0.0003	0.0003	0.001
Gamma	0.99	0.99	0.99
Buffer_size	NULL	10^6	10^6
Batch_size	64	256	100
Max_step	800	800	800
Timestep	10^6	10^6	10^6
Episode	10000	10000	10000

#### Relevant parameters of artificial potential field

**FIGURE 7:**
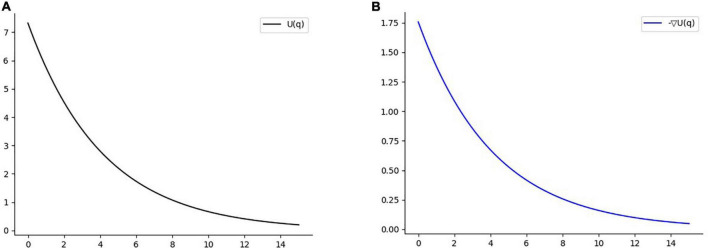
**(A)** Function diagram of repulsion field. **(B)** Negative gradient function diagram of repulsion potential field.

In the attractive potential field, with the attractive potential field gain coefficient *k*_*att*_ = 2 and the potential energy gradient constant, the range of potential energy reward for each cycle of the agent from the attractive potential field is [0,1]. At this time, the reward value obtained by the agent at each timestep does not change dramatically with the distance of the target point. In the repulsion potential field, the influence range of obstacle repulsion is set as ρ_0_ = 15, the repulsion parameter α_*rep*_ = 0.2, and β_*rep*_ = 0.24. At this point, it can be seen from Figure 7 that the potential energy of the repulsion field changes more gently to prevent the phenomenon of sparse reward. Since the agent step size is 1, the scope of punishment brought to the agent within the scope of influence is about[0, 1.56], and the scope of punishment is equivalent to that of reward.

## Experiment and analysis

### Experimental environment design

To verify the feasibility of the improved algorithm, this paper generates an experimental environment with two training environments and two testing environments through the Gym of OpenAI.

#### Training environment

The first training environment is a 300 × 300 square blank closed area. The edge of the environment is set to an untouchable obstacle. There is no obstacle inside the square, which can be freely passed by the agent. There is only a circular target area with a radius of 5. The center position of the target is randomly set in each round. The purpose of this environment training is to make the agent learn to find the shortest path to the target area.

**FIGURE 8:**
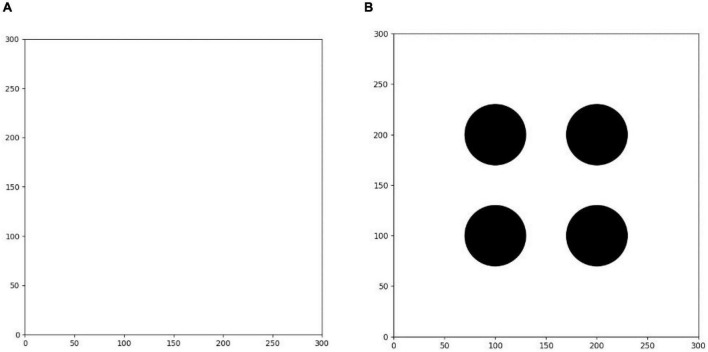
**(A)** Training environment I. **(B)** Training environment II.

As shown in Figure 8, the second training environment is a square closed area of 300 × 300. The edge of the environment is also set to an untouchable obstacle, with four circular obstacles with radius of 30 and a circular target with radius of 5. The positions of four circular obstacles are (100, 100), (100, 200), (200, 200), and (200, 100), respectively. The positions of circular targets are randomly set in each round. Based on the first environment training, the agent is trained in the second environment. The learning task in the environment is more difficult than that in the first environment. The agent finally learns to avoid obstacles correctly and find the shortest path to the target point safely.

### Testing environment

**FIGURE 9:**
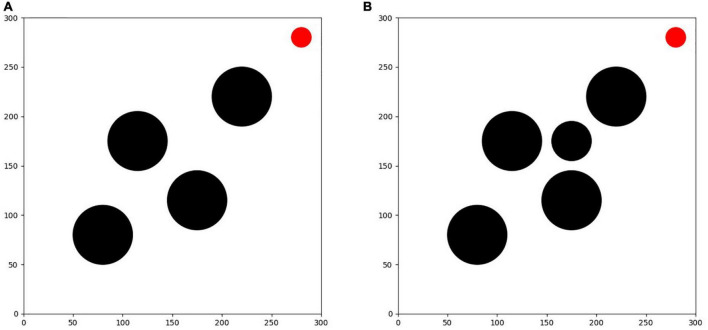
**(A)** Testing environment I. **(B)** Testing environment II.

As shown in Figure 9, the first testing environment is equipped with 4 circular obstacles and 1 circular target with different distribution conditions from the training environment, but with the same shape and size to verify the generalization and feasibility of the trained algorithm. Among them, the circular locations of the obstacles are (80,80), (220,220), (115,175) and (175,115), and the circular location of the target is (280, 280).

The second testing environment is an unfamiliar environment with a sudden threat source. This environment is built based on testing environment I, including 5 black circular obstacles and 1 red circular target, among which 4 obstacles and the target have exactly the same shape, size and location as in testing environment I, all of which are preset in the environment before the test, in addition to 1 emergent obstacle that is added temporarily during the test process. After the testing experiment I can calculate the optimal flight route of each algorithm in the unfamiliar testing environment I, and based on this route information, the emergent obstacle is added to the place where it can have an obstructive effect on each route during the test process, and it is required that the avoidance of the obstacle at this location cannot exceed the constraint limit of the UAV. Therefore, this paper is set to a black circular threat source area with a circle center located at (175, 175) and a radius of 20. The role of this environment is mainly to test the emergency obstacle avoidance capability of each optimization algorithm in response to the emergent threat source. The difficulty of this environment is more enhanced than that of testing environment I, which can provide a better verification of the online planning capability of the algorithms and requires the UAV to have not only the ability to sense the environment in real-time and the real-time planning capability of the route, but also a better maneuvering capability.

## Training experiment

In the training experiment, the agent trains 100,000 timesteps in each training environment. In the training round, the environment will give a small reward value according to the quality of each step of the agent; at the end of each round, if the task is completed, a greater positive reward is given, and if the task fails, a greater punishment is given. When the agent encounters the following situations, it means to complete a round:

• To reach the target, when the agent reaches the target area within the maximum number of steps (max_step), it means to complete the task successfully.
• Beyond the range, when the number of running steps of the agent exceeds, it indicates that the maximum range exceeds the UAV, and the task fails.
• When the agent collides with the environmental boundary or the preset obstacle, the UAV crashes due to collision and the task fails.

In this paper, the average success rate and average reward value are used to evaluate the training of the algorithm. Since the reward value of the reinforcement learning algorithm fluctuates greatly among the single steps, the moving average method is used to smooth the curve in 50 rounds, and the final training curve is output for comparison.

**FIGURE 10:**
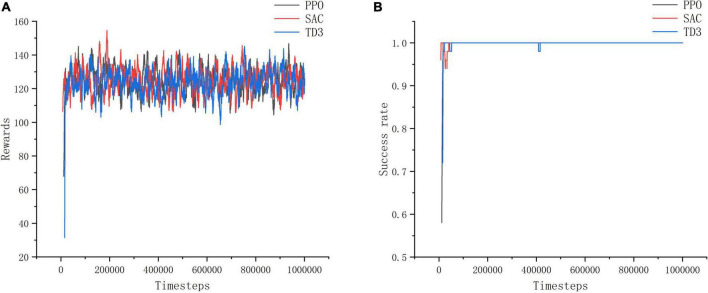
Training environment I indicator change chart. **(A)** Change of reward value in training environment I. **(B)** Change of success rate in training environment I.

From the reward value and success rate change curves in Figure 10, it can be seen that the improved PPO, SAC and TD3 algorithms converge quickly and have better stability in the training environment I, and the differences in the reward value and success rate obtained from convergence are not significant, which proves that all three improved algorithms can perform the task of seeking better in the open environment. Among them, the stability of convergence of the improved PPO algorithm is relatively good with the least curve fluctuation, while the improved SAC algorithm is less stable at the very beginning. This is because the SAC algorithm is a maximum entropy reinforcement learning algorithm, which causes a relatively lower success rate in the initial stage in order to increase the exploration of the environment. However, in the long-term sense, increasing the exploration of the environment at the initial stage of training is more beneficial for the algorithm to find excellent actions and make the trained model have better performance.

**FIGURE 11:**
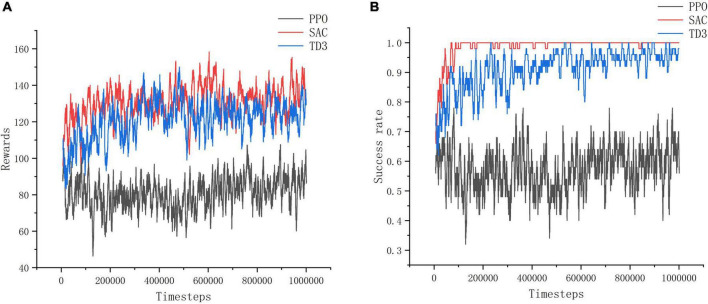
Training environment II indicator change chart. **(A)** Change of reward value in training environment II. **(B)** Change of success rate in training environment II.

As shown in Figure 11, in training environment II, the improved SAC algorithm achieves better convergence in 10w timesteps with minimal curve fluctuations, and its convergence speed and stability are much better than those of the improved PPO algorithm and the improved TD3 algorithm. Among them, the training effect of the improved PPO algorithm is the worst, and the reward value and success rate of its model do not change significantly before and after training in training environment II, the reward value is about 80, and the success rate is about 0.6. The convergence speed of the improved TD3 algorithm is slower than that of the improved SAC algorithm, and it takes about 20w timesteps to reach the convergence effect, and the effect is unstable and fluctuates more, and the reward value and success rate obtained are smaller than those of the improved SAC algorithm is smaller. It can be concluded that the PPO algorithm and TD3 algorithm are less reliable than the SAC algorithm in complex and difficult environments, which is due to the SAC algorithm focuses on the exploration of the action, avoids the problem of overestimation of the action, and can learn more superior experience in complex environments.

Through the experiment, it can be seen that PPO algorithm is more suitable for simple training tasks, and the training effect for complex tasks is poor, while SAC algorithm has the best training effect and can better deal with such tasks.

### Testing experiment

Each algorithm is trained to load the model completed by training, and then put into the testing environment for experiments. PPO, SAC, and TD3 algorithm were tested for 10000 times, and the test results of each algorithm in an unfamiliar environment were counted.

#### Evaluation indicators

Four experimental statistical indicators were set for each group of test experiments: average reward value, average success rate, average path smoothness, and average path length. The calculation methods of each indicator are as follows.

a. Average success rate *M*_*s*_

The average success rate is a key index to measure the generalization and stability of the algorithm as shown in Equation 27. The higher the average success rate of the algorithm in a strange environment, the better the reliability and generalization of the algorithm, and vice versa.*N* is the total number of experiments. It can be calculated as


(25) $$M_{s}=\frac{N_{s}}{N}\times 100\%.$$

b. Average reward value *M*_*r*_

The average reward obtained by the agent after reaching the target point is used to judge whether the algorithm optimizes the track. The higher the reward value is, the better the track is theoretically, and vice versa. *N*_*s*_ is the number of successful planning in the test results. The equation can be written as


(26) $$M_{r}=\frac{\sum R_{i}}{N_{s}}.$$

c. Average path smoothness *M*_*a*_

The less the number of UAV maneuvers, the smaller the turning angle, the lower the requirements for the UAV control system, and the better the track. This index is the average value of the sum of the absolute values of the successful round of UAV tasks. A is the action value of each timestep of UAV in the successful round. The equation can be written as


(27) $$M_{a}=\frac{\sum \sum_{i=1}^{n}\left|a_{i}\right|}{N}.$$

d. Average track length *M*_*l*_

Since the flight speed of the UAV is constant, the track length of the UAV is proportional to the flight time. The average flight time when the task is successful is counted, and then the average track length is calculated. Among them, for the successful round of UAV flight path length. The equation can be written as


(28) $$M_{l}=\frac{\sum L}{N_{s}}.$$

e. Average planning time *M*_*t*_

This index calculates the average planning time of each flight trajectory when the task is successful to test the real-time performance of the algorithm. The equation can be written as


(29) $$M_{t}=\frac{T_{e\,n\,d}-T_{s\,t\,a\,r\,t}}{N_{s}}.$$

#### Experiment with an unfamiliar fixed obstacle environment

**FIGURE 12:**
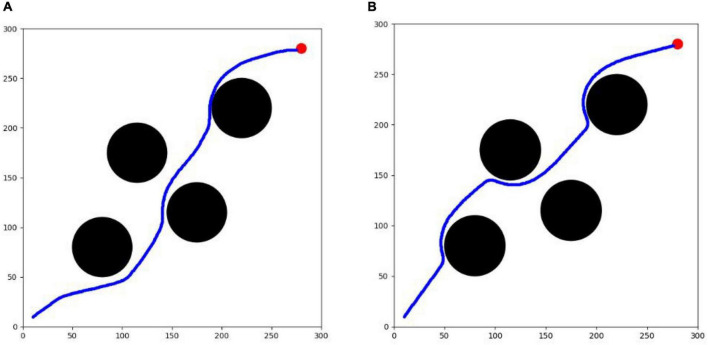
UAV path map. **(A)** SAC algorithm. **(B)** TD3 algorithm.

The optimization results of each algorithm in the testing environment are shown in Table 2, and the UAV route is shown in Figure 12. PPO algorithm cannot complete the task in the testing environment. SAC algorithm and TD3 algorithm can complete the task. The success rate of the two algorithms is 100 %, which can better complete the task. However, the maneuvering amplitude of SAC algorithm is smaller. It can also be seen from the figures that the track obtained by SAC algorithm is smoother, and the total path length is relatively shorter. The average calculation speed of single track of the two algorithms is only 0.02 s, which proves that the calculation speed of the two algorithms is equivalent and can meet the real-time requirements of UAV online track planning. The test results show that SAC algorithm has better effect and stronger generalization in track planning in unfamiliar testing environment.

**TABLE 2:** Comparison of test data.

Algorithm	*M*_*s*_	*M*_*r*_	*M*_*a*_	*M*_*l*_	*M*_*t*_
PPO	0%				
SAC	100%	269.26	14.10	410	0.57
TD3	100%	265.83	44.01	421	0.55

From the results of training experiments and test experiments, it can be seen that SAC algorithm has stronger ability in convergence and generalization when dealing with track planning problems, which can meet the real-time requirements of online track planning.

#### Sudden threat environmental experiment

**FIGURE 13:**
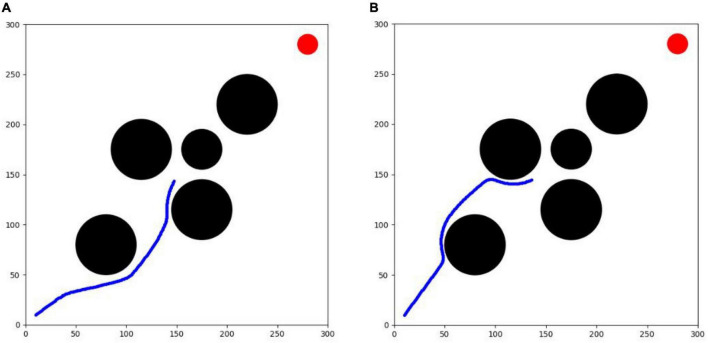
Step 210 2D trajectory map (test II). **(A)** SAC algorithm. **(B)** TD3 algorithm.

From testing experiment I, it can be seen that the horizontal and vertical coordinates of the agent in both algorithms are around 150 when the timestep of the agent is 210. Therefore, a circular threat source with a radius of 2 km is added at (175,175) when the agent in both algorithms is at the 210th timestep, as shown in Figure 13.

After 10,000 test experiments in testing environment II, the results are shown in Table 3. The improved TD3 algorithm could not complete this experiment, while the improved SAC algorithm still has better obstacle avoidance and real-time maneuvering capability in the case of sudden threats. The average success rate of the improved SAC algorithm decreases from 100 to 96% due to the sudden appearance of close circular obstacles, but still maintains a high success rate. To perform obstacle avoidance, the agent has an extra 0.8 increment of maneuver value, the metric increases from 14.1 to 14.9, and the average trajectory length changes from 410 to 413.68, which is equivalent to an average increase of 3-4 timesteps. The planning time for each trajectory is 0.59, which can still meet the real-time requirements of online route planning for UAVs.

**TABLE 3:** Comparison of metrics in testing environment II.

Algorithm	*M*_*s*_	*M*_*r*_	*M*_*a*_	*M*_*l*_	*M*_*t*_
TD3	0%				
SAC	96.0%	268.22	14.90	413.68	0.59

## Conclusion

In this paper, according to the real-time characteristics of UAV navigation tasks, the deep reinforcement learning algorithm is improved combined with the idea of the artificial potential field algorithm. The environmental potential energy information is introduced into the state space, and the potential field difference is introduced into the reward function. The convergence speed of the algorithm is accelerated under the guidance of the potential energy information, and the problem of sparse reward in the reinforcement learning algorithm is solved. The complex experimental tasks are decomposed by course learning, which reduces the difficulty of task learning. Compared with the experimental results of PPO, SAC, and TD3 algorithms, SAC algorithm has faster convergence speed, better path smoothing effect and more superiority in solving this problem.

## Data availability statement

The raw data supporting the conclusions of this article will be made available by the authors, without undue reservation.

## Author contributions

YZ, JS, XZ, HH, and HS wrote part of the manuscript. YZ, JS, and HH analyzed the results and prepared the figures and tables. All the authors contributed to the article and approved the submitted version.
